# Structural and Magnetoresistive Properties of Nanometric Films Based on Iron and Chromium Oxides on the Si Substrate

**DOI:** 10.1186/s11671-016-1684-2

**Published:** 2016-10-20

**Authors:** Aleksey B. Smirnov, Serhii B. Kryvyi, Sergii A. Mulenko, Maria L. Sadovnikova, Rada K. Savkina, Nicolaie Stefan

**Affiliations:** 1 V. Lashkaryov Institute of Semiconductor Physics, National Academy of Sciences of Ukraine, 41 Nauky Ave., Kyiv, 03028 Ukraine; 2 G. Kurdyumov Institute for Metal Physics, National Academy of Sciences of Ukraine, 36 Vernadsky Blvd, Kyiv, 03142 Ukraine; 3 National Institute for Lasers, Plasma and Radiation Physics, 409 Atomistilor Street, Măgurele, PO Box MG-36, Bucharest, 077125 Romania

**Keywords:** Magnetoresistivity, Nanometric films, Transitional metal oxides, 75.70.Ak, 75.50.Ee, 75.47.Lx, 75.50.Pp

## Abstract

Ultraviolet photons of KrF laser (248 nm) was used for the synthesis of nanometric films based on iron and chromium oxides (Fe_2_O_3 − *X*_(0 ≤ *x* ≤ 1) and Cr_3 − *X*_O_3 − *Y*_(0 ≤ *x* ≤ 2; 0 ≤ *y* ≤ 2)) with variable thickness, stoichiometry, and electrical properties. Film deposition was carried out on the silicon substrate Si < 100 > at the substrate’s temperature *T*_S_ = 293 K. X-ray diffraction and X-ray reflectometry analysis were used for the obtained structure characterization. Such a combined investigation reveals the composition and texture for samples investigated and provides useful information about layer thickness and roughness. Fe_2_O_3 − *X*_(0 ≤ *x* ≤ 1) nanometric films demonstrate the negative magnetoresistance in magnetic fields up 7 kOe. At the same time, for hybrid systems of the alternate layers Fe_2_O_3 − *X*_(0 ≤ *x* ≤ 1)/Cr_3 − *X*_O_3 − *Y*_(0 ≤ *x* ≤ 2; 0 ≤ *y* ≤ 2), the positive magnetoresistance as well as the magnetic hysteresis and magnetoresistivity switching effect in the low magnetic fields were observed.

## Background

As we know, the functional characteristics of thin films depend on their architecture and thickness. During the past several years, a great interest has been focused on nanometric films, to test the advantages of reduced thickness in the performances of electronic devices [1]. In particular, a great attention has been paid to nanometric multiferroic materials [2–4]. Control of electric properties of such materials can be possible by using a magnetic field. A close coupling of magnetization and polarization via magnetoelectric and magnetodielectric effects holds promise for new generations of storage media with both magnetic and electric polarization and opens the possibility of electrically reading/writing magnetic memory devices.

In our previous work [5–9], it was shown that thin films based on silicides and oxides of the transitional metals formed by the pulsed laser deposition (PLD) and by the reactive pulsed laser deposition (RPLD) are quite suitable materials for thermo-tenso sensors. The reactive pulse laser deposition is one of the attractive methods for the nanometric film synthesis. The advantages of this method are effectivity, simplicity, environmental safety, and deposition of the layers with precise thicknesses on the different substrates from the various chemical precursors. In other words, the RPLD method application in combination with the material selecting allows creating sensors with required parameters [5–7]. The iron oxide thin films with different sensing properties for thermo-photo-chemical sensors operating at moderate temperature were demonstrated. In the present work, we report on the studies of the magnetoresistive properties of nanometric films of iron and chromium oxides (Fe_2_O_3 − *X*_(0 ≤ *x* ≤ 1), Cr_3 − *X*_O_3 - *Y*_(0 ≤ *x* ≤ 2; 0 ≤ *y* ≤ 2) as well as hybrid structure of the alternate layers Fe_2_O_3 − *X*_(0 ≤ *x* ≤ 1)/Cr_3 *− X*_O_3 − *Y*_(0 ≤ *x* ≤ 2; 0 ≤ *y* ≤ 2) synthesized on silicon substrates by RPLD.

## Methods

It is notable, that oxides synthesized by RPLD techniques have the different stoichiometry as a rule (Fe_2_O_3 − *X*_(0 ≤ *x* ≤ 1), Cr_3 − *X*_O_3 − *Y*_(0 ≤ *x* ≤ 2; 0 ≤ *y* ≤ 2), which depends on laser parameters and the ambient conditions (such as a number of the emitted impulses *N*, temperature substrate *T*_S_, gas pressure in the chamber). Nanometric chromium and iron oxide layers as well as their combination (Fe_2_O_3 − *Y*_/Cr_3 − *X*_O_3 − *Y*_/Fe_2_O_3 − *Y*_/Cr_3 − *X*_O_3 − *Y*_) were grown by RPLD techniques in the vacuum stainless steel reactor on the boron-doped (100)-oriented *p*-type silicon wafers at the substrate’s temperature *T*_S_ = 293 K. The detailed parameters of the all samples are presented in the Table 1.

**Table 1:** Some parameters of typical samples investigated, *T =* 296 K

	Sample composition	*R*_0_, kOhm	Thickness obtained by		Roughness, nm		
					(Si - film)	(film - air)	*R*_a_, (AFM)
			Profilometer, (nm)	XRR (nm)			
#1	Fe_2_O_3 − *X*_, (0 ≤ *x* ≤ 1)	47.0	80 ± 4	73 ± 1	1.4	0.9	0.939
#2	Cr_3 − *X*_O_3 − *Y*_, (0 ≤ *x* ≤ 2; 0 ≤ *y* ≤ 2)	512.0	55 ± 2.75	–	–	–	1.092
#3	Fe_2_O_3 − *Y*_/Cr_3 − *X*_O_3 − *Y*_/Fe_2_O_3 − *Y*_/Cr_3 − *X*_O_3 − *Y*_	22.0	50 ± 2.5	–	–	–	–
#4	Fe_2_O_3 − *Y*_/Cr_3 − *X*_O_3 − *Y*_/Fe_2_O_3 − *Y*_/Cr_3 − *X*_O_3 − *Y*_	7.0	10	10.8 ± 0.5	1.4	1.2	–

The experiment setup is completely described in [5]. Before each deposition, the reactor was evacuated down to a residual pressure of ~4.5 · 10^−5^ Pa to avoid contamination. A pure (99.5 %) Fe and/or Cr target was ablated with a KrF excimer laser pulses (*λ* = 248 nm; *τ*_imp_ = 20 ns) at a fluence of 4.0 J/cm^2^ and frequency repetition rate of 10 Hz in the flow of pure oxygen (99.999 %) which was supplied at the different pressures: 0.1, 0.5, and 1.0 Pa. The number of laser pulses was varied from *N* = 4000 to 6000 depending on the oxygen pressure in the reactor. The target was rotated with frequency ~3 Hz to obtain a smooth ablation procedure. Before each deposition, the target surface was cleaned using 3000 laser pulses with a shutter shielding the substrate. The thickness of the films (*d*) was controlled by “Tensor Instruments” model “Alpha-step 100” profilometer with an accuracy of 5 %.

The X-ray diffraction analysis (XRD) of the samples was realized by standard methods on the X-ray diffractometer “Stoe” at 45 kV and 33 mA (CuK_α_ irradiation) [4, 5]. In addition, X-ray reflectometry (XRR) studies were carried out on high-resolution X-ray diffractometer PANalitical X-Pert PRO MRD using CuK_α1_ characteristic radiation. The CuK_α1_ radiation with a wavelength of 0.15406 nm was separated out using a four-bounce (440) Ge monochromator. The incident X-ray beam was collimated to 0.1 mm gap. The analysis of the measured XRR curves was carried out in the program PANalitical Reflectivity, which is based on the Parratt’s equation [10]. Such a combined investigation not only reveals the composition and texture for samples investigated but also provides useful information about layer thickness, density, and roughness. This information can be used to tune these materials.

Magnetic properties of the sample investigated were studied at room temperature using a vibrating sample magnetometer (LDJ-9500) with a maximum magnetic field of 10 kOe. During the measurements, an external field was applied in the film plane and normal to film plane. Magnetoresistance (MR) measurements were performed by Van der Pown technique on square-shape samples at the room temperature in the magnetic field range up to 7 kOe which was applied normal to film plane. The samples investigated were rigidly mounted in the experimental setup. The point indium electrodes were coated on the film surface in the corner of the square with the side *l* = 1 mm. Linearity of the contacts was tested by the current-voltage characteristic. Exploitation of the low magnetic fields’ range was stipulated by the low value of the charge carriers’ mobility for materials under study (*μ*_*n*_ ≤ 10^−4^ m^2^/V⋅s, [11]). We used an electromagnet as an external magnetic field source. The measurement method allows changing both magnetic field direction and a current (no more than 100 μA) polarity through the sample.

## Results and Discussion

**Fig. 1:**
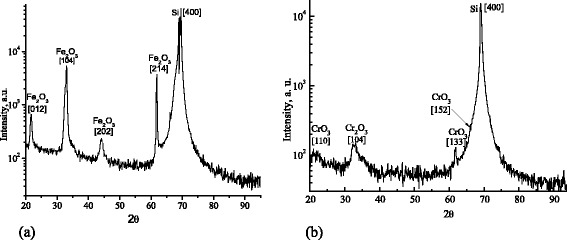
XRD spectra of nanometric films deposited by RPLD on Si substrate. **a** Sample #1 Fe_2_O_3 − *X*_. **b** Sample #2 Cr_3 − *X*_O_3 − *Y*_

Figure 1 shows the XRD pattern of the nanometric films deposited by RPLD on Si substrate. The characteristic halfwidth of peaks and presence of symmetric reflections indicates the polycrystalline structure of nanometric films investigated. The analysis confirms the presence of the transitional metal oxides phase *α*-Fe_2_O_3_ (see Fig. 1a) and Cr_2_O_3_, CrO_3_ (see Fig. 1b). The XRD pattern reveals also the Si (400) diffraction peak that confirms (100) orientation of the silicon substrate.

**Fig. 2:**
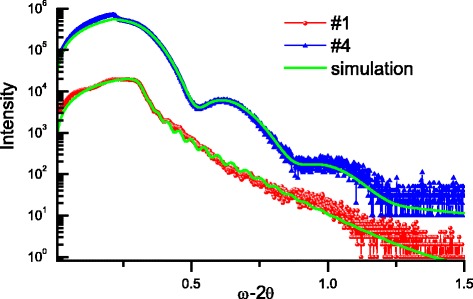
X-ray reflectivity curves of Fe_2_O_3 − *X*_ (sample #1) film and Fe_2_O_3 − *Y*_/Cr_3 − *X*_O_3 − *Y*_ multilayer (sample #4) deposited on Si substrate. *Green lines* show simulations

X-ray reflectivity analysis allowed determining the thickness and roughness of the samples investigated (see Table 1). Interference fringes are created by the phase difference between X-rays reflected from different surfaces. Because of this, the distance between the fringes is inversely proportional to the thickness of the layer. We can see this on Fig. 2. Thicker films Fe_2_O_3 − *X*_ (sample #1) have smaller fringes compared to thinner films Fe_2_O_3 − *Y*_/Cr_3 − *X*_O_3 − *Y*_ (sample #4). It is necessary to note a good correlation between the thicknesses of the samples investigated by XRR and by profilometer (see Table 1). Moreover, the roughness value of the samples investigated is amounted ~1.4 nm that shows a good quality of the finishing characteristics of nanometric films deposited by RPLD method.

**Fig. 3:**
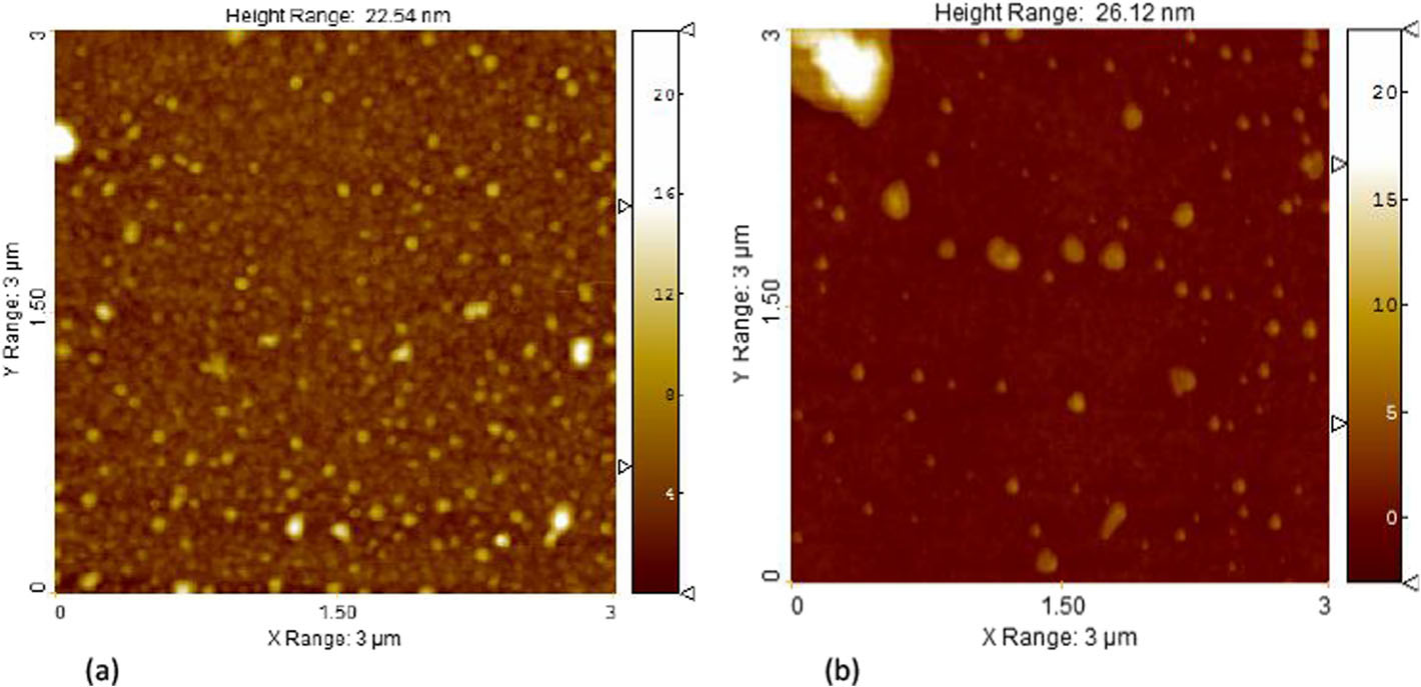
AFM images of **a** Fe_2_O_3 − *X*_ nanometric film obtained at 0.5 Pa by 5000 laser pulses, *R*_max_ = 22.846 nm. **b** Cr_3 − *X*_O_3 − *Y*_ nanometric film obtained at 0.5 Pa by 4000 laser pulses, *R*_max_ = 26.084 nm

AFM topographic measurements allow one to monitor the morphological distinction between nanometric films of iron and chromium oxides. The AFM images of typical samples investigated are shown in Fig. 3. Closely packed pseudospherical grains ranging from 40 to 70 nm in dimensions are visible on the surface of Fe_2_O_3 − *X*_ film (Fig. 3a). Some grains are distinguished by a height. The root-mean-square roughness over a surface fragment 3 × 3 μm^2^ in area amounts to 1.522 nm. At the same time, on the background of a flat surface of Cr_3 − *X*_O_3 − *Y*_ film the freestanding grains ranging from 30 to 140 nm in dimensions are observed (Fig. 3b). Accordingly, the relief became more developed, and the root-mean-square roughness over a surface fragment 3 × 3 μm^2^ in area increased to 2.221 nm.

The magnetization measurements have shown that the Fe_2_O_3 − *X*_ film exhibits ferromagnetic behavior with a remanent magnetization (Mr) of 0.16 memu and coercivity (Hc) of 0.26 kOe. The saturation magnetization field is larger when the applied field is perpendicular to film plane that demonstrate the shape anisotropy of Fe_2_O_3 − *X*_ films. The hysteresis loop did not reach magnetization saturation, even at the maximum applied magnetic field (to 10 kOe). The magnetization of Cr_3 − *X*_O_3 − *Y*_ films is practically absent even at a very high applied magnetic field. The structure of the alternate layers Fe_2_O_3 − *X*_(0 ≤ *x* ≤ 1)/Cr_3 − *X*_O_3 − *Y*_(0 ≤ *x* ≤ 2; 0 ≤ *y* ≤ 2) also exhibits ferromagnetic behavior with a very small hysteresis loop. Moreover, saturation magnetization Mrs (0.4 memu) of the multilayer structure considerably less than Mrs (1.6 memu) of Fe_2_O_3 − *X*_ film.

**Fig. 4:**
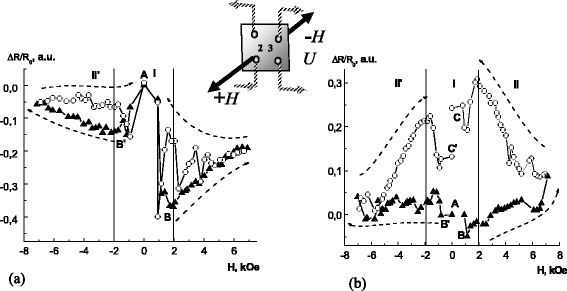
The dependencies of the magnetoresistance *ΔR*/*R*(*H*). **a** For sample #1, *black triangle* - increasing magnetic field; *white circle* - diminishing magnetic field. **b** For sample #3, *black triangle* - increasing magnetic field; *white circle* - diminishing magnetic field

Magnetoresistance was estimated by using following equation: *ΔR*/*R*_0_ *=* (*R*(*H*) − *R*_0_)/*R*_0_ [12], where resistance *R = R*_12,34_ *= U*_34_ */I*_12_ according to Ohm’s low. See inset in Fig. 4 with the contact numbering. Figure 4 shows the field dependences of the MR obtained for samples #1 and #3 at the room temperature. First of all, it can be clearly seen the negative magnetoresistance for sample #1 (see Fig. 4a). The dependence *ΔR*/*R*_0_(*H*) is characterized by the non-monotonic decrease of MR with the minimum at ± 2 kOe (points B, B’ on Fig. 4a). At the same time, the positive magnetoresistance with non-monotonic feature on the reverse branch (the *ΔR/R* maximum at ± 2 kOe) characterizes the multilayer structure (see Fig. 4b). The hysteresis-like behavior is typical for both nanometric structures. It was found that the magnetoresistivity comes back into the initial point on the reverse branch of the hysteresis loop for sample #1 (point A, Fig. 4a) and does not come back for sample #3 (points A and C (C’), Fig. 4b). A non-monotonic feature at ±2 kOe points out to the magnetoresistivity “switching” effect in both samples.

As it is known, the polycrystalline *α*-Fe_2_O_3_ and Cr_2_O_3_ under normal conditions crystallize in the lattice with *R3c* space group symmetry [13]. At the same time, their magnetic properties are determined by Neel temperature (*T*_N_ *α*-Fe_2_O_3_ = 955 K [13]; *T*_N Cr__2_O_3_ = 307 K [14]), i.e., these materials have a different magnetic structure.

Thermodynamically, hematite (α-Fe_2_O_3_) is the most stable in the family of iron (III) oxides: (*α*-Fe_2_O_3_, *β*-Fe_2_O_3_, maghemite (*γ*-Fe_2_O_3_), and *ε*-Fe_2_O_3_). The d-d transitions and metal charge transfer play important roles in tuning the *n*-type semiconducting band gap of hematite. This material exhibits soft ferromagnetism between 260 K and the Neel temperature. Hematite shows interesting properties like high photochemical stability, low-toxicity, and suitable redox potential for photocatalytic water dissociation. The Cr_2_O_3_ compound has an eskolaite-like structure. It is one of the most important wide band gap (*E*_g_ ≈ 3 eV) *p*-type semiconductor transition metal oxide material. This kind of *p*-type wide band gap oxide semiconductors may be a good candidate material for UV light emitter using nanolasers and optical storage system. In references, many crystalline modifications of chromium oxides such as rutile (CrO_2_), CrO_3_, CrO_4_, corundum (Cr_2_O_3_), Cr_2_O_5_, and Cr_5_O_12_ have been reported. Among these modifications, Cr_2_O_3_ is the most stable magnetic-dielectric oxide material. It is antiferromagnetic up to *T*_N Cr__2_O_3_ = 307 K and magnetoelectric (ME) – material in which magnetic and electric order coexist.

It should be supposed that the features of the *ΔR*/*R*(*H*) dependences of the nanometric films investigated are connected with the magnetic properties of the original materials. In other words, the hematite demonstrates a soft ferromagnetism, i.e., a mixed antiferromagnetic-ferromagnetic state. This is so-called *canted antiferromagnetism*, when in the unit cell of hematite the four magnetic ions’ vectors are directed non-strictly antiparallel but at the angle *φ* [15]. As it is known, the behavior of ferromagnetic materials at low magnetic fields is describes by the Rayleigh law $\varDelta M={\chi}_H\cdot \varDelta H+\frac{1}{2}\cdot \eta \cdot \varDelta {H}^2$, where *ΔM* is a magnetizations’ changes, Δ*H* is changes of the magnetic field intensity, *η* is the Rayleigh constant describing the irreversible processes, and *χ*_*H*_ is a magnetic viscosity describing the reversible part of magnetization. Thus, a hysteresis-like and non-monotonic behavior of magnetoresistivity of the samples investigated is a result of the soft ferromagnetism in *α*-Fe_2_O_3_.

The magnetic symmetry of the Cr_2_O_3_ allows existing of the direct and indirect magnetoelectric (ME) effect [15–17]. The direct ME effect causes the additional electrical polarization in the sample under the magnetic field influence. It is obvious that the observable “memory” effect for the sample #3 containing the chromium oxide, when *ΔR/R* does not comes back into the initial point at diminishing magnetic field, can be associated with magnetoelectric properties of the one.

## Conclusions

Ultraviolet photons of KrF laser (248 nm) was used for the synthesis of nanometric films based on iron and chromium oxides (Fe_2_O_3 − *X*_(0 ≤ *x* ≤ 1) and Cr_3 − *X*_O_3 − *Y*_(0 ≤ *x* ≤ 2; 0 ≤ *y* ≤ 2)) with variable thickness, stoichiometry, and electrical properties. Film deposition was carried out on the silicon substrate Si < 100 > at the substrate’s temperature *T*_S_ = 293 K. Based on X-ray diffraction and X-ray reflectometry analysis, the obtained structure characterization was carried out. Such a combined investigation reveals the composition and texture for samples investigated and provides useful information about layer thickness and roughness. The roughness value of the samples investigated is amounted ~1.4 nm that shows a good quality of the finishing characteristics of nanometric films deposited by RPLD method. Fe_2_O_3 − *X*_(0 ≤ *x* ≤ 1) nanometric films demonstrate the negative magnetoresistance in magnetic fields up to 7 kOe. At the same time, for hybrid systems of the alternate layers Fe_2_O_3 − *X*_(0 ≤ *x* ≤ 1)/Cr_3 − *X*_O_3 − *Y*_(0 ≤ *x* ≤ 2; 0 ≤ *y* ≤ 2), the positive magnetoresistance as well as the magnetic hysteresis and magnetoresistivity switching effect in the low magnetic fields were observed. Up to now, Cr_2_O_3_ has been most promising material for realistic applications close to room temperature in ME-controlled spintronic elements like MERAM. Besides, the use of the (Cr_1 - *x*_Fe_*x*_)_2_O_3_ structures will expand the fields of spintronic application if the ME properties of Cr_2_O_3_ do not get lost [18]. Thus, the hybrid system of the alternate nanometric layers Fe_2_O_3 − *X*_(0 ≤ *x* ≤ 1)/Cr_3 − *X*_O_3 − *Y*_(0 ≤ x ≤ 2; 0 ≤ *y* ≤ 2) obtained by our technology can be used as multi-parameter magnetic sensors operating at the moderate temperature.

## Abbreviations

ME: Magnetoelectrical effect
PLD: Pulsed laser deposition
RPLD: Reactive pulsed laser deposition
XRD: X-ray diffraction
XRR: X-ray reflectometry
MERAM: Magnetoelectric Random Access Memory

## References

[1] Nalwa HS (2000). Handbook of nanostructured materials and nanotechnology.

[2] Kimura T, Goto T, Shintani H, Ishizaka K, Arima T, Tokura Y (2003). Magnetic control of ferroelectric polarization. Nature.

[3] Hur N, Park S, Sharma PA, Ahn JS, Guha S, Cheong S-W (2004). Electric polarization reversal and memory in a multiferroic material induced by magnetic fields. Letters Nature.

[4] Hemberger J, Lukenheimer P, Fichtl R, Krugvon Nidda H-A, Tsurkan V, Loidl A (2005). Relaxor ferroelectricity and colossal magnetocapacitive coupling in ferromagnetic CdCr_2_S_4_. Letters Nature.

[5] Caricato AP, Luches A, Romano F, Mulenko SA, Kudryavtsev YV, Gorbachuk NT, Fotakis C, Papadopoulou EL, Klini R (2007). Deposition of thin films for sensors by pulsed laser ablation of iron and chromium silicide targets. Appl Surf Sci.

[6] Mulenko SA, Gorbachuk NT, Stefan N (2014). Laser synthesis of nanometric iron oxide films with high Seebeck coefficient and high thermoelectric figure of merit. Lasers Manufacturing Materials Process.

[7] Mulenko SA, Gorbachuk NT, Stefan N (2014). Laser synthesis of nanometric chromium oxide films with high Seebeck coefficient and high thermoelectric figure of merit. Int Res J Nanosci Nanotechnol.

[8] Mulenko SA, Petrov YN, Gorbachuk NT (2012). Photon synthesis of iron oxide thin films for thermo-photo-chemical sensors. Appl Surf Sci.

[9] Cariacato AP, Luches A, Martino M, Valerini D, Kudryavtsev YV, Korduban FV, Mulenko SA, Gorbachuk NT (2010). Deposition of chromium oxide thin films with large thermoelectromotive force coefficient by reactive pulsed laser ablation. J Optoelectron Adv Mater.

[10] Parratt G (1959). Surface studies of solids by total reflection of X-rays. Phys Rev.

[11] Folen VJ (1970). Landolt-Bornstein magnetic and other properties of oxides and related compounds III/4b.

[12] Кuchis EV (1974). Methods of investigating the Hall effect.

[13] Grygar T, Bezdička P, Dĕdeček J, Petrovsky E, Schneeweiss O (2003). Fe_2_O_3_-Cr_2_O_3_ system revised. Ceramics – Silikáty.

[14] Sahoo S, Mukherjee T, Belashchenko KD, Binek C (2007). Isotermal low-field tuning of exchange bias in epitaxial Fe/Cr_2_O_3_/Fe. Appl Phys Lett.

[15] Morrish AH (1994) Canted antiferromagnetism: hematite. World Scientific Publishing Co. Pte. Ltd, Singapore

[16] Pisarev RV, Krichevtsov BB, Pavlov VV (1991). Optical study of the antiferromagnetic-paramagnetic phase transition in chromium oxide Cr_2_O_3_. Phase Transit Multinational J.

[17] Pyatakov AP, Zvezdin AK (2012). Magnetoelectric and multiferroic media. Phys–Usp.

[18] Kleemann W (2013). Magnetoelectric spintronics. J Appl Phys.

